# Spacetime Imaging of Group and Phase Velocities of Terahertz Surface Plasmon Polaritons in Graphene

**DOI:** 10.1021/acs.nanolett.4c04615

**Published:** 2025-01-02

**Authors:** Simon Anglhuber, Martin Zizlsperger, Eva A. A. Pogna, Yaroslav A. Gerasimenko, Anastasios D. Koulouklidis, Imke Gronwald, Svenja Nerreter, Leonardo Viti, Miriam S. Vitiello, Rupert Huber, Markus A. Huber

**Affiliations:** † Regensburg Center for Ultrafast Nanoscopy (RUN) and Department of Physics, University of Regensburg, 93040 Regensburg, Germany; ‡ Istituto di Fotonica e Nanotecnologie, Consiglio Nazionale delle Ricerche (CNR-IFN), 20133 Milano, Italy; § NEST, CNR-Istituto Nanoscienze and Scuola Normale Superiore, Piazza San Silvestro 12, 56127 Pisa, Italy

**Keywords:** terahertz surface plasmon polaritons, near-field optical microscopy, s-SNOM, field-resolved, graphene, time-resolved, hypertemporal map, phase velocity, group velocity, all-optical control

## Abstract

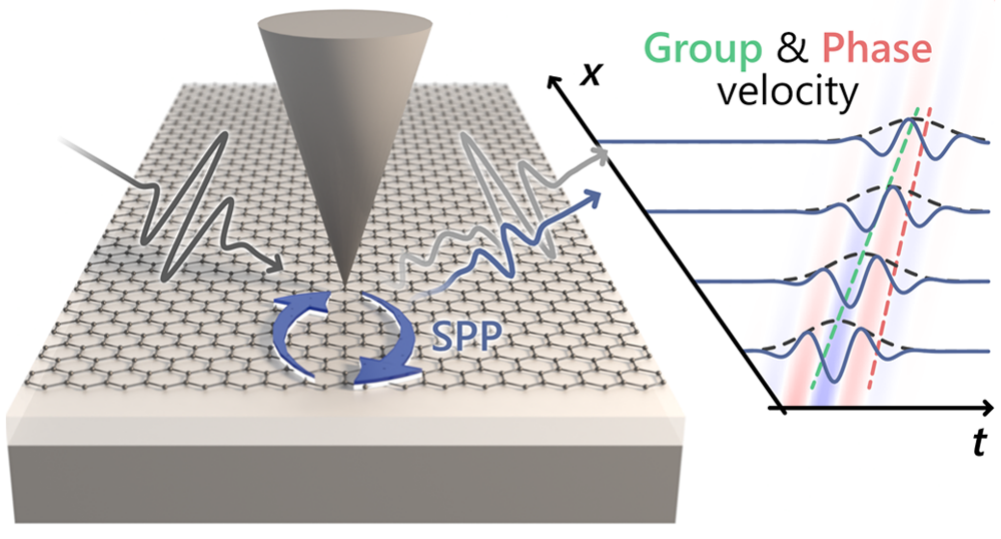

Detecting electromagnetic radiation scattered from a tip–sample junction has enabled overcoming the diffraction limit and started the flourishing field of polariton nanoimaging. However, most techniques only resolve amplitude and relative phase of the scattered radiation. Here, we utilize field-resolved detection of ultrashort scattered pulses to map the dynamics of surface polaritons in both space and time. Plasmon polaritons in graphene serve as an ideal model system for the study, demonstrating how propagating modes can be visualized and modeled in the time domain by a straightforward mathematical equation and normalization method. This novel approach enables a direct assessment of the polaritons’ group and phase velocities, as well as the damping. Additionally, it is particularly powerful in combination with a pump–probe scheme to trace subcycle changes in the polariton propagation upon photoexcitation. Our method readily applies to other quantum materials, providing a versatile tool to study ultrafast nonequilibrium spatiotemporal dynamics of polaritons.

Tailoring light-matter interaction in confined modes at the surface of materials, so-called surface polaritons, has been a long-standing research topic.^1^ In the mid-infrared spectral range, plasmon polaritons in graphene have attracted significant attention^2,3^ owing to strong field confinement and sensitivity to surrounding materials, making it ideal for sensors and detectors,^4−6^ waveguides,^7^ and polaritonic logic devices.^8^ Near-field microscopy has proven to be a valuable tool in measuring these polaritons directly in real space, since the sharp metallic tip provides the necessary *k*-vectors to excite and detect polaritons.^9−11^ Mid-infrared near-field studies on photoexcited dynamics^12−15^ of polaritons have also been reported.^16−18^ Furthermore, it has been shown that the hybridization of graphene plasmon polaritons with surface modes in specifically tailored gold nanostructures increased their confinement also in the terahertz (THz) frequency range.^19−23^ So far, most studies rely on the frequency domain to describe the polariton dispersion using pseudoheterodyne^9,10^ or interferometric^18^ measurement techniques. However, it has recently been shown that imaging propagating modes directly in the time domain can lead to novel insights in the polariton properties in the low-frequency THz spectral range.^24,25^

Here, we build on this idea and investigate low-frequency surface plasmon polaritons (SPPs) in graphene directly in the time domain. Using a straightforward normalization method, the pure polariton propagation can be visualized and the SPP damping as well as the group and phase velocities can be extracted. We extend the study by comparing dry-exfoliated graphene samples with graphene grown by chemical vapor deposition (CVD). The time-resolved real-space data sets can also be used to extract the phase velocity and the nontrivial propagation direction of polaritons for each spatial position within a 10 × 6 μm^2^ area with different scattering geometries, which is particularly relevant for the investigation of nanoscale inhomogeneities and in-plane anisotropy. Finally, we show how spatiotemporal mapping can directly trace the nonequilibrium dynamics of polaritons following ultrafast photoexcitation with sub-polariton-cycle precision.

Our approach makes use of the conventional technique of echolocation detection of polaritons, enhanced by the feature of directly resolving the scattered electric field using electro-optic sampling (EOS, Figure 1a). We couple THz pulses to a sharp metallic tip of a scattering-type scanning near-field optical microscope (s-SNOM, the system is detailed in refs (26−28)), placed in close proximity to the sample, leading to an instantaneous scattering response, *E*_scat_^inst^, that depends on its local dielectric function ε. The tip sets the volume of interaction, i.e. the nanometric spatial resolution, and provides the finite momentum required to launch SPPs that propagate from the tip apex position (Figure 1a, sketched orange arrow). After being reflected at the dielectric boundary, the SPPs reach the tip apex position again, leading to an additional scattering signal, *E*_scat_^SPP^, with a time delay that depends on the tip’s distance to the boundary. The superposition of the instantaneous and the SPP response is coupled out into the far field, where the complete waveform of the scattered electric field *E* is detected in the time domain using EOS.^29^ To suppress contributions of the far-field background, the tip is operated in tapping mode and the scattering signal is demodulated at the *j*^th^ harmonic of the tapping frequency *f*_tip_.

**Figure 1:**
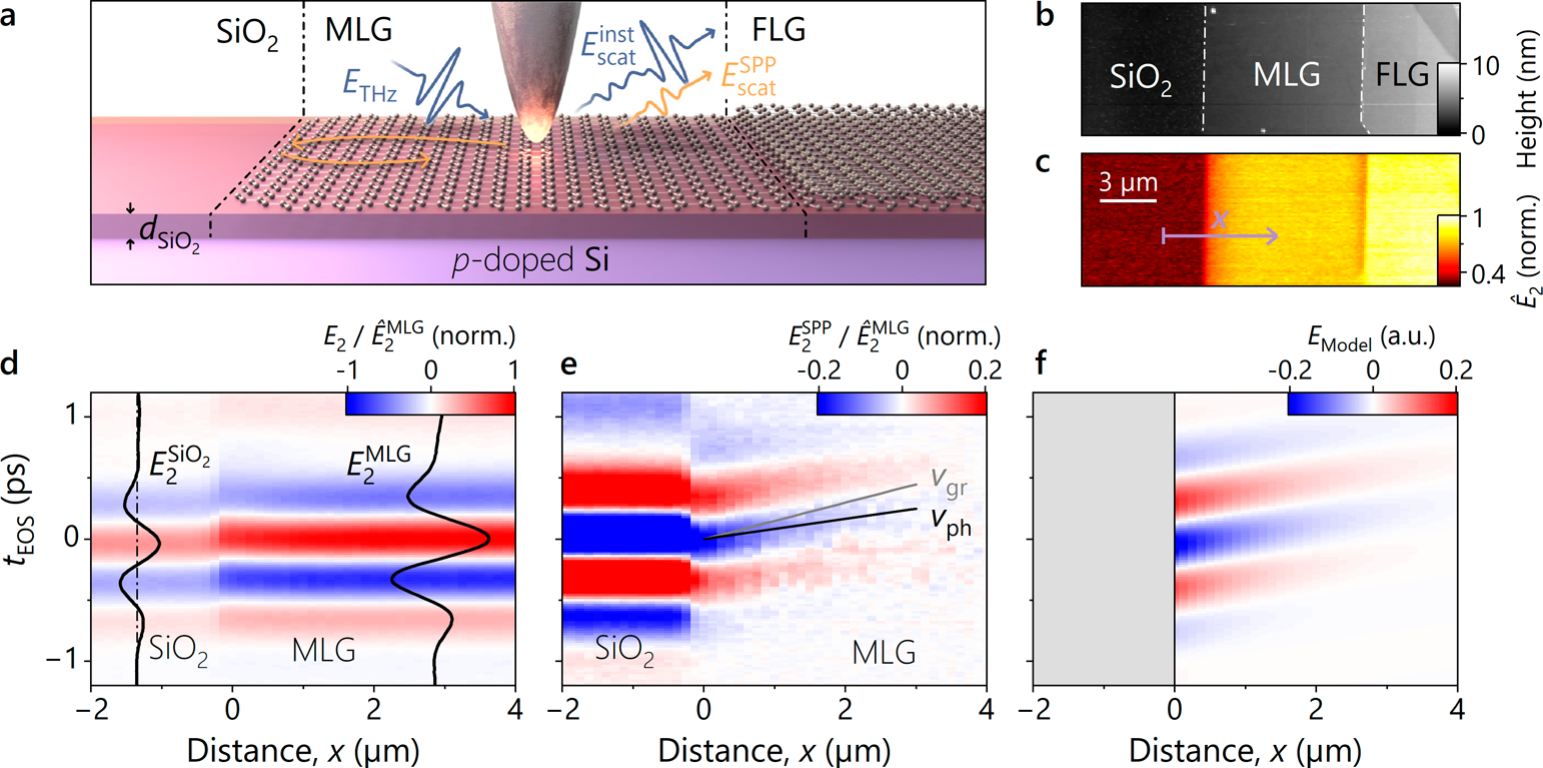
Time-domain imaging of surface plasmon polariton propagation in dry-exfoliated graphene. a) Schematic illustration of time-domain retrieval of surface plasmon polaritons (SPPs) in graphene using THz near-field microscopy. A THz pulse (*E*_THz_) is coupled into evanescent near fields at the tip apex, launching SPPs in the graphene sample, which are reflected at dielectric boundaries (orange arrows). The scattering response consists of two contributions: an instantaneous response (*E*_scat_^inst^) and a delayed response owing to SPP propagation (*E*_scat_^SPP^). The investigated high quality graphene samples are prepared by dry exfoliation, yielding clean regions of monolayer (MLG) and few-layer graphene (FLG), as well as the pristine substrate. The latter consists of a Si wafer with a layer of SiO_2_ of thickness *d*_SiO_2__ = 300 nm. b) Atomic force microscopy image of regions of the substrate, partially covered by MLG and FLG. c) Corresponding map of the peak of the scattered THz waveform *Ê*_2_. On MLG, the signal decreases monotonically with decreasing distance to the edges. d) Hypertemporal map obtained by moving the tip along a line across the SiO_2_/MLG interface (magenta arrow in c). Each vertical linecut corresponds to a scattered THz waveform *E*_2_, recorded at a specified distance to the interface, *x*. The two black waveforms serve as a reference. *E*_2_^SiO_2_^ represents the waveform at the dash-dotted line and *E*_2_^MLG^ is the reference transient recorded at *x* = 4 μm. e) Difference of the hypertemporal map in d and the reference waveform recorded on MLG (*E*_2_^MLG^), revealing the bare SPP propagation in MLG with different values for the phase and group velocities (gray and black lines). While the phase velocity can be extracted by tracing a maximum or a zero-crossing of the carrier wave, the group velocity corresponds to the speed at which the envelope of the wave packet travels. f) Propagation of the SPP wave in e, described mathematically through eq 1 with *v*_ph_ ≈ 24.1 μm/ps, *v*_gr_ ≈ 13.3 μm/ps, *f*_SPP_ ≈ 1.31 THz, ϕ ≈ 3.57 rad, σ ≈ 419 fs, *A*_SPP_ ≈ 0.22, and *δ*_*x*_ ≈ 2.31 μm, as determined from a global fit of all transients (SI, Section 4).

In a first step, we investigate the surface of a mechanically dry-exfoliated graphene flake on a Si wafer with a 300-nm-thick buffer layer of SiO_2_ (see Supporting Information (SI), Section 1, for details on the fabrication) featuring regions of monolayer graphene (MLG) and few-layer graphene (FLG). Scanning the tip on the dry-exfoliated sample simultaneously yields the topography (Figure 1b) and a map of the peak of the waveform of the second order (*j* = 2) scattered electric field, *Ê*_2_ (Figure 1c). The scattering response on the MLG (FLG) region is drastically increased by a factor of 2.4 (2.7) compared to the SiO_2_/Si substrate, proving the strong interaction of the THz radiation with graphene. Overall, we see a homogeneous response in the center of each individual region, which indicates a high surface quality of the samples.

Interestingly, the peak scattered near-field response *Ê*_2_ shows a monotonic decrease moving from the center of the graphene flake toward both edges, which we take as a clear indication of a propagating polariton mode. To prove this hypothesis, we record full waveforms of the scattered electric field *E*_2_, i.e. the scattered transients, while moving the tip across the SiO_2_/MLG interface (magenta arrow in Figure 1c). In this hypertemporal line scan (Figure 1d), the signal recorded on graphene consists of both the instantaneous response, *E*_2_^MLG^, and the scattering contribution from the propagating SPP, *E*_2_^SPP^. Therefore, only the latter depends on the distance, *x*, to the SiO_2_/MLG interface. To eliminate the instantaneous response and isolate the signature of the propagating SPP (Figure 1e), we subtract a reference transient (Figure 1d, *E*_2_^MLG^) recorded at *x* = 4 μm. This is far away from any edges, and hence we do not expect any contribution from the reflected SPP, which would affect the result (SI, Section 2). In the resulting referenced hypertemporal map, we clearly observe a shift of the waveforms to later EOS delay times, *t*_EOS_, for increasing distance from the edge of the graphene flake, which is among the most direct ways to observe propagating polaritons (see SI, Section 2, for an extended scan across the MLG/FLG interface). The time shift between neighboring waveforms is on the order of 10 fs (SI, Section 3). Remarkably, the propagation speed of the electric field envelope (determined by the group velocity, gray line in Figure 1e) is different from the evolution of the zero-crossings (determined by the phase velocity, black line in Figure 1e). The full evolution of the polaritons’ electric field can be captured in a single mathematical equation:
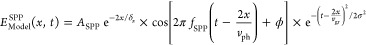
1which contains the group velocity *v*_gr_, the phase velocity *v*_ph_, the decay length *δ*_*x*_, the center frequency *f*_SPP_, the initial amplitude *A*_SPP_, the temporal width σ, and the carrier envelope offset phase ϕ. It should be noted that the amplitude decay is purely exponential without any additional factor for the geometrical decay of the mode, which typically needs to be included when operating at tip-edge distances larger than the polariton wavelength due to the isotropic, circular energy spreading as a function of time.^18,30^ Here, we are in the near-field limit of the polariton, measuring the field profile at distances less than one polariton wavelength from the edge, where geometrical optics is not yet applicable.^25^

For appropriate values of the parameters, an analytical map of the spacetime propagation can be drawn (Figure 1f). All transients from *x* = 0 to 3 μm in Figure 1e are used to determine the limited set of fitting parameters of the equation above (SI, Section 4), i.e. , , *f*_SPP_ ≈ 1.31 THz, ϕ ≈ 3.57 rad, σ ≈ 419 fs, *A*_SPP_ ≈ 0.22, and *δ*_*x*_ ≈ 2.31 μm (Figure 2a). The extracted velocities have a direct correspondence in the polariton dispersion (Figure 2b), yielding the position in frequency ω_SPP_ = 2*π f*_SPP_ and momentum *k*_SPP_ = *ω*_SPP_/*v*_ph_ ≈ 0.34 μm^-1^, as well as the local slope  of the dispersion in the probed frequency range. The experimentally extracted SPP wave vector *k*_SPP_ corresponds to a confinement factor of  and a SPP wavelength of λ_SPP_ ≈ 18 μm. The polariton damping is captured by *δ*_*x*_, which is only a fraction of the polariton wavelength () due to the strong damping of the THz SPP mode at room temperature, without encapsulation in e.g. hexagonal boron nitride.^19^

**Figure 2:**
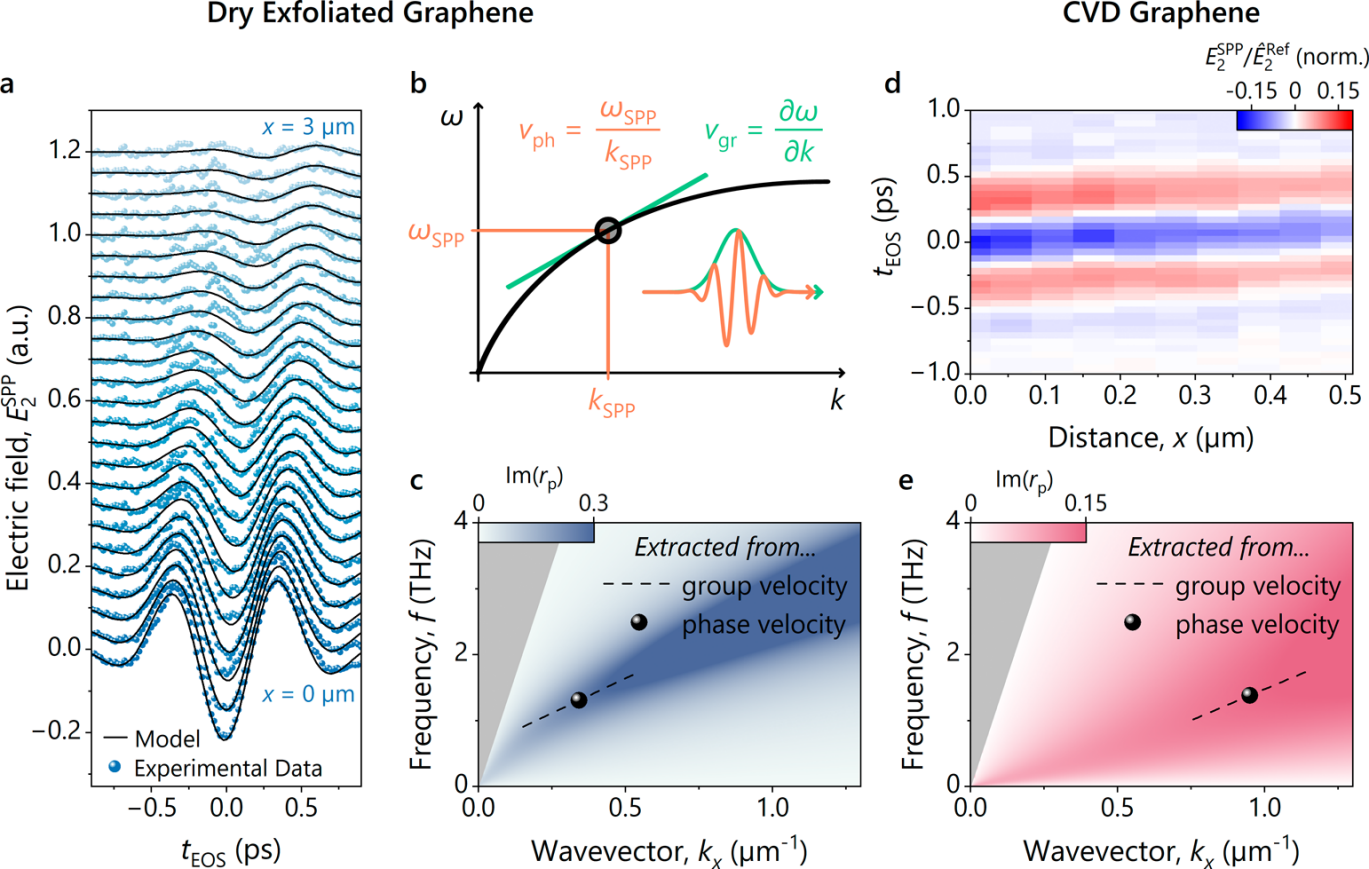
Tailoring surface plasmon polaritons by material design. a) Experimentally extracted electric field of the SPPs *E*_2_^SPP^ on dry-exfoliated graphene as a function of EOS delay time *t*_EOS_ (blue spheres) for different distances *x* to the MLG/substrate interface (see Figure 1e). The full evolution of the SPP wave packet can be perfectly described by eq 1 (black lines). b) Phase and group velocities in nonlinear dispersion relations. The phase velocity, , represents the speed at which the carrier wave travels, while the group velocity, , describes the propagation speed of the wave packet’s envelope. By knowing both phase and group velocity, one can determine a point of the dispersion relation and its gradient at the center frequency of the wave packet. c) Simulated dispersion relation of the multilayer sample system (graphene parameters: *E*_F_ = 183 meV, τ = 150 fs). The imaginary part of the Fresnel coefficient *r*_p_(*f*, *k*), calculated by using the transfer matrix method, reveals a SPP mode (blue background). The black sphere and line correspond to the experimentally determined points of the dispersion relation and its gradient at the center frequency of the SPP wave packet. d) Referenced hypertemporal map of a graphene sample fabricated via chemical vapor deposition (CVD). e) Numerically calculated dispersion relation for the precharacterized CVD-grown graphene sample (*E*_F_ = 230 meV). The assumed lower scattering time (τ_CVD_ = 23 fs) results in a more heavily smeared out dispersion relation of the SPPs. Applying the same analysis as with the dry-exfoliated graphene sample yields the marked point of the dispersion relation with its respective local gradient.

To corroborate these experimental findings, we compare the extracted dispersion relation with the numerically calculated one of the full multilayer sample stack by employing the transfer matrix method.^31^ For this purpose, we describe the graphene sheet through its optical conductivity,^32^ given by  with Fermi energy *E*_F_ = 183 meV and scattering time τ = 150 fs, in agreement with a Raman characterization of a similar sample (SI, Section 5) and literature values for exfoliated graphene.^33,34^ The substrate is modeled using the dielectric function of SiO_2_, ε_SiO_2__ = 4.4 (ref (35)), with a thickness of *d*_SiO_2__ = 300 nm, followed by an infinite half-space of Si, characterized by a dielectric constant of ε_Si_ = 11.7 (ref (36)) and a Drude susceptibility with a doping density of *n*_Si_ = 3 × 10^15^ cm^–3^. The resulting imaginary part of the Fresnel coefficient *r*_p_ in Figure 2c clearly displays the SPP dispersion relation in graphene and perfectly agrees with the experimentally extracted values (black sphere and dashed line). The role of the Fermi energy, scattering time, and substrate doping on the calculated dispersion relation are discussed in SI, Section 6.

Furthermore, we perform the same extraction for a graphene sample grown via CVD with a precharacterized *E*_F_ ≈ 230 meV (SI, Section 5) and reportedly lower scattering times.^37^ The scattering rate appears to be dominated by defects within the sample rather than surface residues from the fabrication process (see SI, Section 7, for a comparison before and after vacuum annealing). From the spatiotemporal maps (Figure 2d), we extract a smaller 1/e-decay length, *δ*_*x*_ = 1.4 μm, than in exfoliated graphene (*δ*_*x*_ = 2.3 μm, Figure 1e). Additionally, lower phase and group velocities are found, i.e. . The results are consistent with a numerical simulation that predicts a SPP mode featuring stronger damping and a less strongly curved dispersion relation in the accessible spectral range (Figure 2e; *E*_F_ = 230 meV, τ_CVD_ = 23 fs). The same analysis is applied to a CVD graphene flake with a higher Fermi energy (*E*_F_ ≈ 430 meV), where a higher phase velocity of  is retrieved, in excellent agreement with the numerical model (SI, Section 8).

As a result, our approach offers a simple way of extracting the propagation velocities and decay lengths of SPPs in different samples even in strongly damped cases, at room temperature, without the requirement for a cryogenic environment, a complex analysis in the frequency domain, or a specific sample shape. In principle, our time-domain analysis (eq 1) could also capture a modification of the polariton dispersion during propagation, but, in our samples this effect did not contribute significantly, which is why we kept the temporal width σ constant.

Proceeding beyond polariton propagation in one dimension, our technique allows us to even visualize the spatiotemporal evolution in a full femtosecond snapshot movie. Here, we choose the CVD-grown sample, which features more topographic structures and thus provides more complex edge geometries for the reflection of polaritons (Figure 3). We scan a region with two main features where polaritons can reflect: a rupture in the center and a larger hole on the left side of the scan. We record three snapshots of the scattered THz electric field as a function of tip position for different *t*_EOS_ and reference the signal to a clean area on graphene far away from any edges (Figure 3a). At the EOS delay time 100 fs before the peak of the reference transient (*t*_EOS_ = −100 fs, top panel), we find a homogeneous increase in signal strength when moving away from the substrate/graphene interfaces along perpendicular trajectories. At later EOS delay times (*t*_EOS_ = −50 fs, central panel; *t*_EOS_ = 0 fs, bottom panel), the width of this region increases, as indicated by the contour lines, signifying the propagation of polaritons. To quantitatively extract the full two-dimensional (2D) propagation of the SPPs, we trace the time delay, *t*_0_, corresponding to the first zero-crossing after the main peak of the graphene reference transient (SI, Section 4). Figure 3b exemplifies this idea by displaying the time value *t*_0_ along a line that crosses the graphene/substrate/graphene interface (blue dashed line in Figure 3a, black line in Figure 3b shows height profile), where a slope of  is found, corresponding to a phase velocity of . By extracting the 2D gradient of the zero-crossings (SI, Section 4), we obtain both the magnitude of the phase velocity *v*_ph_ (Figure 3c) and the direction of the SPP propagation α (Figure 3d) for all positions, represented in false-color images. The magnitude of *v*_ph_ near the graphene/SiO_2_ interface is found to be uniform, with a propagation direction perpendicular to the interface. This straightforward and fast acquisition of *v*_ph_ in arbitrary directions is particularly helpful for the study of anisotropic materials supporting SPPs with nonspherical wavefronts. By determining the motion of the polariton electric field envelope, also the group velocity can be traced (not shown, see SI, Section 4, for extraction details).

**Figure 3:**
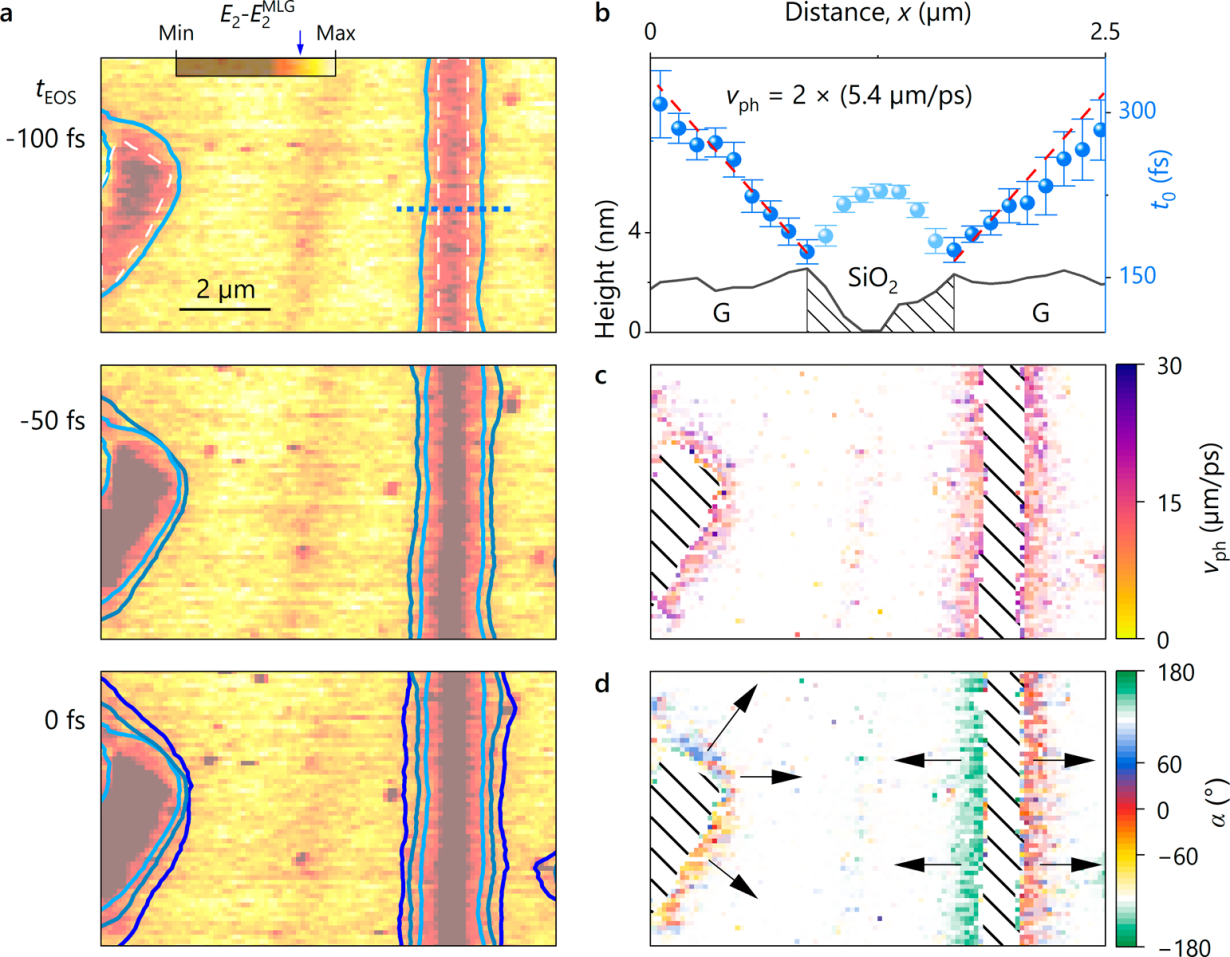
Two-dimensional spatiotemporal videography of propagating SPPs on CVD graphene. a) Femtosecond snapshots of the spatially resolved scattered electric field *E*_2_ for different EOS delay times *t*_EOS_, recorded on CVD graphene and referenced on the bare graphene response *E*_2_^MLG^. The white dashed lines outline the area of the SiO_2_/Si substrate. The blue lines visualize the polariton propagation in time by showing the evolution of one contour line over time (value marked by the blue arrow in the colorbar), after spatially filtering each image using a nonlocal means algorithm. b) Height profile (black line) and temporal position of the first zero-crossing *t*_0_ (blue spheres) after the main maximum of the reference *E*_2_^MLG^ along the blue dashed line in a. On both sides of the SiO_2_ substrate, marked by the black dashed area, a steady increase in the position of the zero-crossing with increasing distance to the edge is observed with a slope of  (red dashed lines). The zero-crossing position *t*_0_ is extracted by fitting a Gaussian wave packet to each data point. The error bars represent the 68% confidence interval of the retrieved *t*_0_ from the fit. c, d) Two-dimensional map of the extracted phase velocity *v*_ph_ (c) and direction of propagation α (d) retrieved by taking the 2D gradient of a map of the zero-crossings *t*_0_ using the Sobel operator. The black arrows serve as a guide to the eye, highlighting the direction of propagation. For a clearer representation in c and d, data points with larger errors in *t*_0_ are displayed with increased transparency, effectively highlighting the confidence in the measurement. Details on the extraction can be found in the SI, Section 4.

Measuring in the time domain also enables us to probe nonequilibrium dynamics of SPPs in a direct way. For this purpose, we utilize ultrashort optical pump pulses (pulse duration, 140 fs; center wavelength, 515 nm; fluence, ) to photoexcite electrons in dry-exfoliated graphene by interband absorption, and isolate the pump-induced changes in the scattering signal by demodulating at the first positive sideband to the *j*^th^ harmonic originating from the pump modulation (Figure 4a, see refs (26, 27)). The pump-induced change Δ*E*_1_ of the scattered electric field *E*_1_ on the center of a graphene flake for a pump delay time of *t*_p_ = 800 fs (blue) reveals that photoexcitation leads to a reduction (Δ*E*_1_ < 0) of the scattering signal compared to the unexcited reference waveform *E*_1_ (dashed gray line), and the magnitude of the change Δ*E*_1_ corresponds only to ∼0.2% of *E*_1_ at *t*_EOS_ = 0 fs. By changing the pump delay, we enable subcycle control of the charge-carrier distribution of graphene (orange waveform; *t*_p_ = −100 fs). To trace these underlying local dynamics upon photoexcitation, we record the peak of the pump-induced signal Δ*Ê*_1_ (*t*_EOS_ = 0 fs) as a function of pump delay time *t*_p_ (Figure 4b).

**Figure 4:**
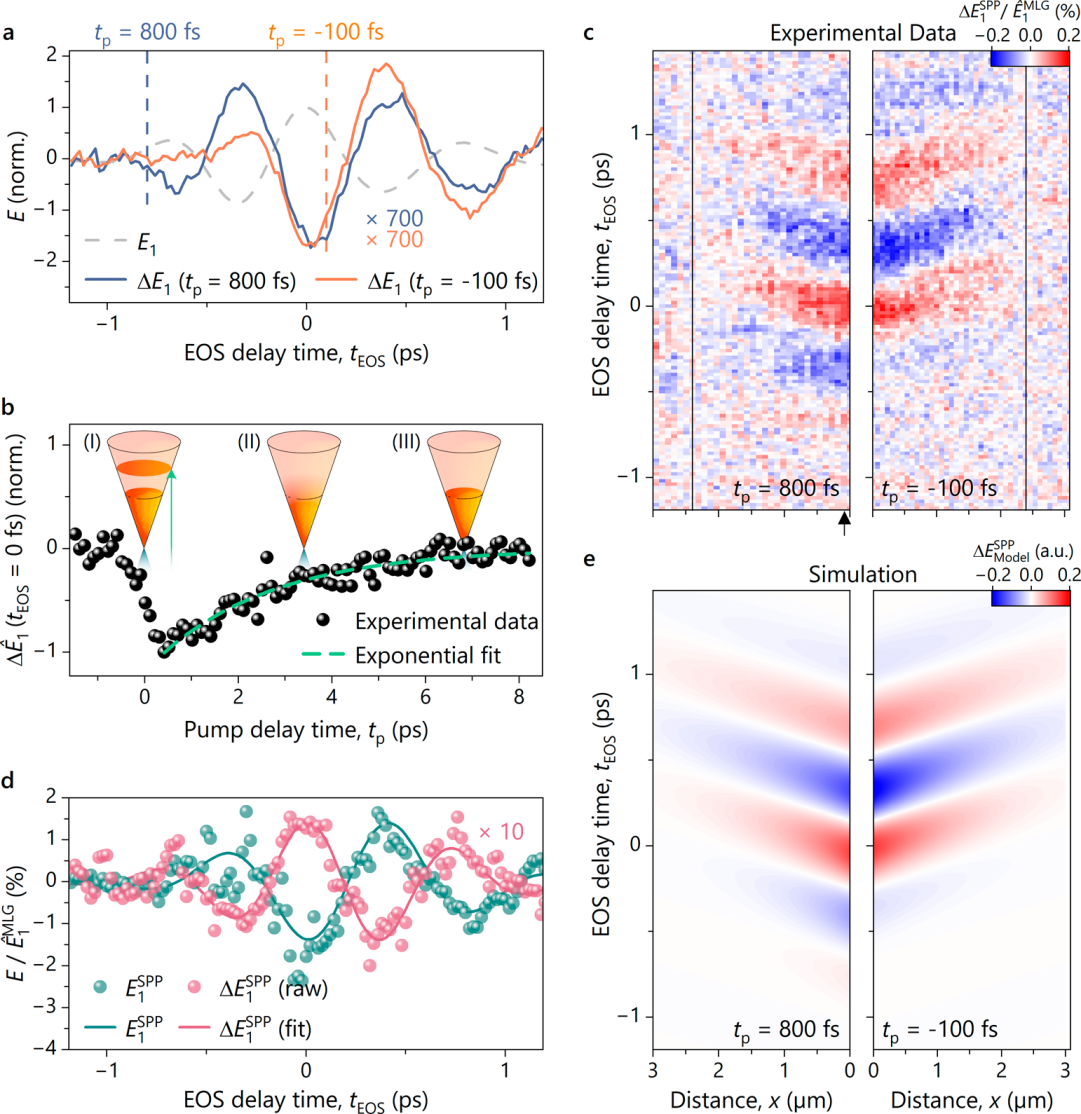
Ultrafast control of surface plasmon polaritons in dry-exfoliated graphene. a) Pump-induced change of the electric field Δ*E*_1_ on graphene for two different pump delay times (blue, *t*_p_ = 800 fs; orange, *t*_p_ = −100 fs) compared to the reference scattered waveform *E*_1_ (dashed gray line) as a function of the EOS delay time *t*_EOS_. b) Peak of the pump-induced signal Δ*Ê*_1_ at *t*_EOS_ = 0 fs as a function of the pump delay time *t*_p_. Photoexcitation creates a nonequilibrium distribution of carriers (Inset I) that rapidly thermalizes through electron–electron scattering, forming a hot Fermi Dirac distribution (Inset II). The pump–probe curve recorded on graphene shows an exponential decay behavior that can be attributed to the relaxation of the hot carriers back to the equilibrium distribution (Inset III). Fitting this curve with an exponential function of the form e^–*t*/τ^ (green dashed line) yields a lifetime τ ≈ 2.5 ps for the excited charge carriers. c) Hypertemporal map of the pump-induced electric field Δ*E*_1_ referenced to a transient at the spatial position of the solid line for two different pump delay times, *t*_p_ = 800 fs (left) and *t*_p_ = −100 fs (right). For both pump delay times, we observe waveforms that shift toward later delay times with increasing distance to the dielectric interface at *x* = 0. Changing the pump delay time allows us to influence the amplitude of already-launched SPPs with subcycle precision. d) Surface plasmon polariton waveform *E*_1_^SPP^ (turquoise) and corresponding pump-induced change Δ*E*_1_^SPP^ (magenta) from the referenced hypertemporal maps right at the interface (black arrow in c). The amplitude of Δ*E*_1_^SPP^ corresponds to 10% of that of *E*_1_^SPP^. The raw data (spheres) are fitted with a cosine function multiplied with a Gaussian envelope for the sake of clarity. e) Simulation of the hypertemporal map shown in c. By adding a time-dependent amplitude with the form of the pump–probe curve (SI, Section 9) shown in b to eq 1, we can reproduce the experimental data displayed in c with great agreement.

Photoexcitation at *t*_p_ = 0 ps creates a nonthermal distribution of carriers (Figure 4b, first inset), leading to a pump-induced signal that sets in within the response time of our experiment (∼400 fs, see ref (26)). This distribution rapidly thermalizes through electron–electron scattering^38^ (<20 fs, not visible) and thus forms a hot Fermi Dirac distribution with a significantly increased electronic temperature compared to room temperature (Figure 4b, second inset), leading to a reduction of the conductivity at THz frequencies.^39,40^ Subsequently, the signal decays within ∼(2.5 ± 0.3) ps to its original 1/e-value, as extracted by an exponential fit (error corresponds to 95% confidence interval). This agrees well with previous studies in the mid-infrared spectral range,^16^ where the relaxation of hot carriers back to the Fermi energy (Figure 4b, third inset) is attributed to scattering with optical and acoustic phonons.^41^

In a final step, we demonstrate subcycle control of the propagation of SPPs in graphene by recording hypertemporal maps across a SiO_2_/MLG boundary for two pump delay times (Figure 4c). We eliminate the instantaneous change in the scattering response caused by the pump pulse by subtracting a reference transient Δ*E*_1_ far away from any edges (solid black lines), and thereby isolate the influence of photoexcitation on the SPP propagation. We observe a similar pattern as in the unpumped case, with decreasing signal strength and a shift of the waveforms to later *t*_EOS_ with increasing distance to the interface. This is expected for a decaying propagating surface mode whose overall amplitude is decreased due to the reduction of the graphene THz conductivity upon photoexcitation. Subcycle control is achieved by tuning the pump delay *t*_p_, where we either affect the whole main cycle of the SPP (*t*_p_ = 800 fs) or inject carriers just within the main cycle (*t*_p_ = −100 fs), influencing only later *t*_EOS_. This is equivalent to a change of the polariton at two different times during its propagation. From our data, we extract the difference between the excited and unexcited case, yielding the relative change to the polariton amplitude upon photoexcitation. To this end, we compare the SPP waveform *E*_1_^SPP^ and the corresponding pump-induced change Δ*E*_1_^SPP^ directly at the flake’s edge at *t*_p_ = 800 fs (Figure 4d) after normalizing to the instantaneous background *Ê*_1_^MLG^. It is evident that photoexcitation reduces the amplitude of the electric field of the polariton corresponding to a change of ∼10% of the overall polariton amplitude.

Finally, we model our measurement using eq 1. The only adjustment needed to describe the experimental data is making the amplitude time-dependent *A*_SPP_ → *A*(*t*) (see SI, Section 9). The resulting analytical maps for the two different pump–probe delays (Figure 4e) show a good agreement with the data. Generally, all parameters of eq 1 can become time-dependent upon photoexcitation, allowing for the extraction of the dynamics of the different material parameters. For example, a bending of the polariton lobes would indicate a change in group or phase velocity, while a change in the damping would result in pump-induced changes with a maximum effect not occurring at the edge of the sample. Microscopically, the scattering time of the polariton in our doping regime is expected to be dominated by scattering with thermal phonons^19,30^ and, hence, only shows a weak variation upon photoexcitation. Therefore, the most straightforward assumption of only a changing amplitude due to a decrease in THz conductivity^39,40^ already accurately represents our experimental results.

In conclusion, we have demonstrated an innovative near-field imaging approach to resolve the propagation of polaritons directly in the time domain using graphene SPPs as a benchmark system. The phase velocity, group velocity, and the damping can be directly extracted from a single hypertemporal map without going to the frequency domain, allowing us to extract information on a sizable portion of the polariton dispersion curve, even in the case of strongly damped polaritons at room temperature. The method also captures minute changes in the polaritons’ properties related to fabrication-dependent material parameters (e.g. *E*_F_ and τ), as we demonstrated by comparing exfoliated and CVD-grown samples. Recording femtosecond snapshots enables us to trace the time evolution of the polariton in two dimensions. Finally, the direct access to the polariton propagation in time allows for unprecedented control of the nonequilibrium polariton propagation. We showed first evidence of photoinduced changes to the polariton properties at different times after launching a polariton. Our work paves the way for the ultrafast study of intriguing polaritonic effects such as Bloch polaritons^42^ or plasmonic amplification by pumping.^43^ Furthermore, it can be easily extended beyond graphene to probe polaritons in a wide range of materials including anisotropic polaritons in α-MoO_3_ and black phosphorus,^18,44^ or surface modes in topological insulators, as well as to alternative spectral ranges where EOS of the scattered electric field can be implemented.^45^

## Supporting Information Available

(1) Sample fabrication; (2) extended hypertemporal scan across a MLG/FLG interface; (3) quantifying the minimal time shifts in the hypertemporal scans; (4) modeling surface plasmon polariton propagation; (5) Raman characterization of graphene samples; (6) impact of Fermi energy, scattering time, and doping of the substrate on the dispersion relation; (7) evaluating the effect of vacuum annealing on SPP propagation in CVD graphene; (8) measurement on a CVD flake with a complementary Fermi energy; (9) modeling nonequilibrium surface polariton propagation (PDF)

## Abbreviations

THz: terahertz
s-SNOM: scattering-type scanning near-field optical microscope
EOS: electro-optic sampling
SPP: surface plasmon polariton
MLG: monolayer graphene
FLG: few-layer graphene
CVD: chemical vapor deposition
2D: two-dimensional
