# Supplementary material for: Spacetime Imaging of Group and Phase Velocities of Terahertz Surface Plasmon Polaritons in Graphene
Source: Nano Lett. 2025 Jan 2;25(6):2125–32. doi: 10.1021/acs.nanolett.4c04615 (PMC11827103; doi:10.1021/acs.nanolett.4c04615)
Supplement: Supplementary file 1 — nl4c04615_si_001.pdf [file nl4c04615_si_001.pdf]

Supporting Information

Spacetime Imaging of Group and Phase Velocities of Terahertz Surface Plasmon Polaritons in Graphene

*Simon Anglhuber,[#1] Martin Zizlsperger,[#1] Eva A. A. Pogna,[2] Yaroslav A. Gerasimenko,[1] Anastasios D. Koulouklidis,[1] Imke Gronwald,[1] Svenja Nerreter,[1] Leonardo Viti,[3] Miriam S. Vitiello,[*3] Rupert Huber,[1] Markus A. Huber[*1]*

[1]Regensburg Center for Ultrafast Nanoscopy (RUN) and Department of Physics, University of Regensburg, 93040 Regensburg, Germany

[2]Istituto di Fotonica e Nanotecnologie, Consiglio Nazionale delle Ricerche (CNR-IFN), 20133 Milano, Italy

[3]NEST, CNR – Istituto Nanoscienze and Scuola Normale Superiore, Piazza San Silvestro 12, 56127 Pisa, Italy

[#]These authors contributed equally.

[*]Miriam S. Vitiello (miriam.vitiello@sns.it), [*]Markus A. Huber (markus.huber@ur.de);

Contents

1. Sample fabrication 2
2. Extended hypertemporal scan across a MLG/FLG interface 2
3. Quantifying the minimal time shifts in the hypertemporal scans 3
4. Modeling surface plasmon polariton propagation 5
5. Raman characterization of graphene samples 6
6. Impact of Fermi energy, scattering time, and doping of the substrate on the dispersion relation ... 8
7. Evaluating the effect of vacuum annealing on SPP propagation in CVD graphene 10
8. Measurement on a CVD flake with a complementary Fermi energy 10
9. Modeling nonequilibrium surface polariton propagation 12

1. Sample fabrication

Large-area monolayer graphene flakes were dry-exfoliated from a HOPG-Z crystal onto the SiO_2/Si substrate using a conventional scotch-tape technique. The substrate was cleaned in acetone and isopropanol, and subsequently treated in oxygen plasma just before the exfoliation. For the exfoliated flake presented in Figure 1, we used a silicon substrate (specific resistivity, $1 - 10\ \Omega\ \text{cm}^{-1}$) with a 300-nm-thin cover layer of SiO_2 and for Figure 4 a Si substrate (specific resistivity, $0.001 - 0.005\ \Omega\ \text{cm}^{-1}$) with a 285-nm-thin cover layer of SiO_2. Both SiO_2 cover layers were fabricated using dry oxidation techniques to reduce the effect of trapped charges in the dielectric.

Large-area monolayer graphene (MLG) grown by chemical vapor deposition (CVD) on copper was transferred onto the SiO_2/Si (intrinsic, resistivity $> 10\ \text{k}\Omega\ \text{cm}^{-1}$) substrate. This was done by using a polymethyl-methacrylate (PMMA, $950\ \text{K}$) membrane, spin coated at $2000\ \text{rpm}$ on the surface of a MLG sample $(1 \times 1\ \text{cm}^2)$ on copper. The PMMA-MLG-Cu sample was then placed floating on a solution of $1\ \text{g}$ of ammonium persulfate and $40\ \text{ml}$ of deionized (DI) water to etch the Cu substrate. Once the Cu etching is complete, the PMMA-MLG film is transferred in a beaker with DI water and lifted with the host SiO_2/Si substrate. This sample is left to dry overnight and the PMMA membrane is chemically removed with acetone. The sample has been precharacterized using Raman spectroscopy yielding a Fermi energy of $\sim 230\ \text{meV}$ (SI, Section 5).

2. Extended hypertemporal scan across a MLG/FLG interface

Figures S1a & b are the extended versions of Figure 1d & e of the main text, displaying the recorded total scattered electric field E_2 (Figure S1a) and the isolated SPP electric field (Figure S1b) as the tip moves across the SiO_2/MLG and MLG/FLG interfaces. The polariton also reflects at the MLG/FLG interface, showing a sloped propagation toward the center of the graphene flake. Interestingly, the reflected polariton from the MLG/FLG interface exhibits an overall lower amplitude as compared to the polariton reflected from the substrate/graphene interface. We suggest that this variation is due to the different dielectric material contrast at the respective boundaries, leading to a different reflection condition.

In general, for the presented analysis, it is important to take the reference transient at a position where the field of the surface polariton has significantly decreased, i.e. far away from any edges. Otherwise, curved traces in the referenced hypertemporal map are obtained together with a strong residual field at positions in the center of the flake (Figure S1c). To improve the signal-to-noise ratio of the subtraction, neighboring transients can be averaged to one reference if they are not influenced by the surface polariton. Throughout the manuscript, we consistently averaged three transients centered around the plotted reference lines, except Figure 4, where five transients were used.

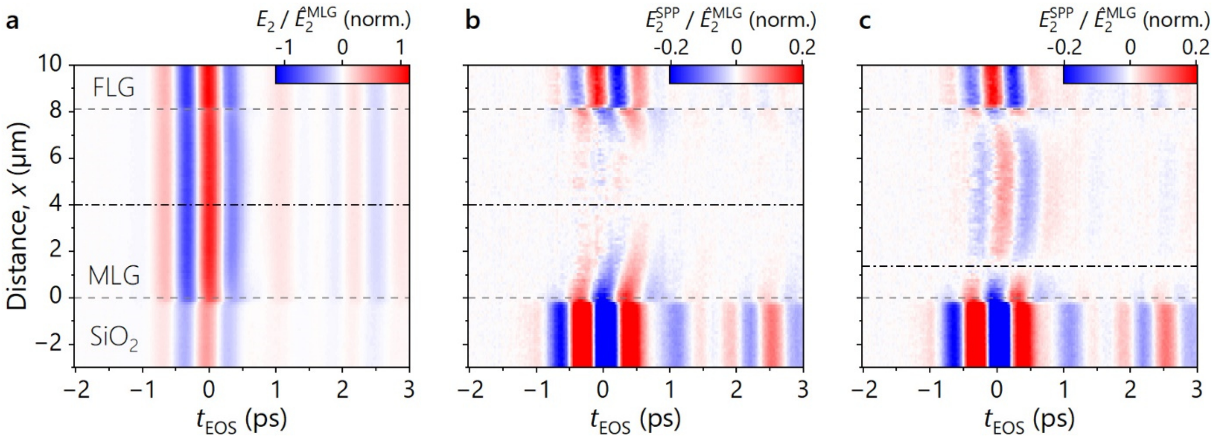

Figure S1. Hypertemporal scan across the substrate/graphene and graphene/few-layer-graphene interface. a) Hypertemporal map across the extended scan range. b) Difference of the hypertemporal map in Figure S1a and the reference waveform recorded at $x = 4$ µm (dash-dotted line). Surface plasmon polaritons emerging from reflection off both the substrate/graphene and the graphene/few-layer-graphene interface are revealed. c) Difference of the hypertemporal map in Figure S1a and a reference waveform recorded at $x = 1.375$ µm, too close to a dielectric boundary. This leads to a characteristic curved shape in the signatures of the referenced hypertemporal map with a strong residual signal in the center of the scan, far away from both interfaces.

3. Quantifying the minimal time shifts in the hypertemporal scans

To quantify the measured time shifts of the waveforms presented in Figure 1e of the main text, we fit all transients individually with the following equation:

$$E_{\text{Model}}^{\text{SPP}}(x,t) = A_{\text{SPP}}(x)\cos\left[2\pi\, f_{\text{SPP}} \times \left(t - t_{\text{ph}}\right)\right] \times \mathrm{e}^{-\frac{\left(t-t_{\text{gr}}\right)^2}{2\sigma^2}},$$

where t_{ph} represents a time shift of the carrier wave and t_{gr} the maximum of the envelope. To obtain the most accurate fitting of the zero-crossings, f_{SPP} and σ are fixed in this section. The fit for the first

ten transients is shown in Figure S2a, also marking the first zero-crossing after the main peak of the carrier-wave as t_0 (red spheres in Figure S2a and plotted as function of distance x in Figure S2b). Finally, the time shift Δt_0 between two neighboring transients can be extracted (Figure S2c), with most values on the order of 10 fs and an accuracy of up to ± 3 fs. The extracted value of $\Delta t_0 \approx 10$ fs is in excellent agreement with the value obtained from the global fit presented in the main text, where $\Delta t_0 = 2\Delta x / v_{\mathrm{ph}} \approx 10.4$ fs. Slight deviations from the general trend are observed for the first two data points, where a smaller value for Δt_0 is retrieved, which we attribute to a remaining influence of the bare substrate to the polariton's electric field very close to the edge**.**

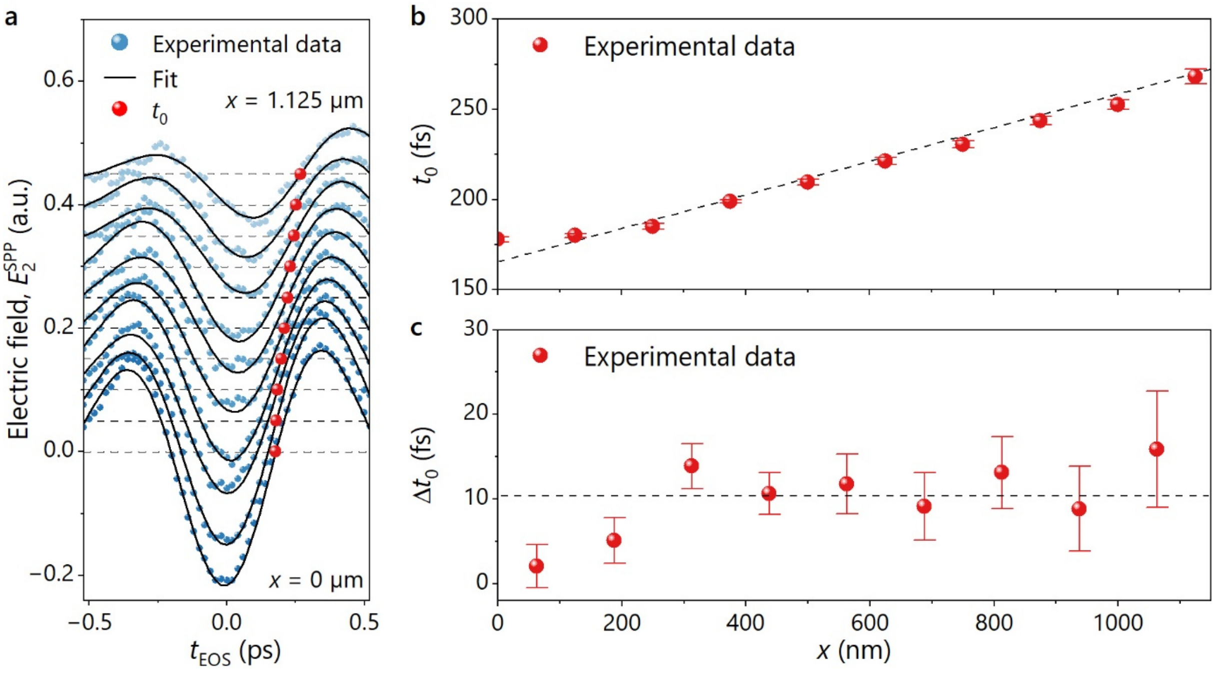

Figure S2. Benchmarking the setup by extracting time shifts. a) Transients from Figure 2a (blue spheres) individually fit with the modified equation as described above (black lines). The red spheres indicate the position of the zero-crossing of the waveform, which shifts to later EOS delay times with increasing distance x from the edge. b) Extracted zero-crossing t_0 from Figure S2a with error bars corresponding to the 68% confidence interval as determined from the fit. The dashed black line is a guide to the eye, indicating that the zero-crossing evolves approximately linear with distance to the edge. c) Difference between two neighboring zero-crossings Δt_0 extracted from b. The horizontal dashed line corresponds to the time-shift between two transients as expected from $\Delta t_0 = 2\Delta x / v_{\mathrm{ph}} \approx 10.4$ fs with $\Delta x = 125$ nm being the distance between the positions of the two transients and $v_{\mathrm{ph}} = 24.1$ µm/ps extracted from the global fit of the main text.

4. Modeling surface plasmon polariton propagation

The parameters describing the spatiotemporal evolution of the polariton are determined by fitting eq 1 to all polariton waveforms recorded for different positions x, simultaneously. This is achieved by minimizing the following scalar function,[27] representing the goodness of the fit for the current input parameters $\vec{p}$ of our model:

$$\sum_x \sum_t \left| E(x,t) - E_{\mathrm{eq}}(x,t,\vec{p}) \right|^2$$

with E the respective datapoint and E_{eq} the solution of eq 1 for the set of input parameters $\vec{p} = (v_{\mathrm{ph}}, v_{\mathrm{gr}}, f_{\mathrm{SPP}}, \phi, \sigma, A_{\mathrm{SPP}}, \delta_x)$. To extract these parameters, we set $x = 0$ to the position of the polariton wave closest to the edge and $t_{\mathrm{EOS}} = 0$ to the maximum of its envelope.

For the two-dimensional analysis presented in Figure 3, the waveform of the isolated SPP for each position is extracted from a set of 20 snapshots recorded at EOS delay times ranging from $t_{\mathrm{EOS}} = -350$ fs to 650 fs (see Figure 3a for three snapshots recorded at $t_{\mathrm{EOS}} = -100$ fs, -50 fs, and 0 fs, respectively). The waveform at each measurement position (x, y) is fit with a Gaussian wave packet as described by

$$E_{\mathrm{fit}}(t) = A_{\mathrm{map}} \times \cos(2\pi \times 1.31\ \mathrm{THz} \times t + \varphi) \times \mathrm{e}^{-\frac{(t-t_{\mathrm{gr}})^2}{2\Gamma^2}},$$

where A_{map} is an amplitude, φ a phase, Γ the standard deviation of the Gaussian envelope, and t_{gr} a time shift in the envelope similar to the description of eq 1 in the main text. For a consistent retrieval of the same zero-crossing t_0 across the whole area, the first zero-crossing after the peak of the reference waveform is used. By calculating the two-dimensional gradient of t_0 for the x (y) direction using the Sobel operator, we retrieve the gradient G_{x,t_0} (G_{y,t_0}). The local phase velocity corresponds to the inverse of its magnitude $v_{\mathrm{ph}} = G^{-1} = \left({G_{x,t_0}}^2 + {G_{y,t_0}}^2\right)^{-1/2}$ and the direction of propagation can be calculated by $\alpha = \mathrm{atan2}\left(G_{y,t_0}, G_{x,t_0}\right)$, where atan2 is the 2-argument arctangent. A map of the group velocity and the corresponding direction of propagation can be obtained using the same approach, substituting t_0 with t_{gr}.

The transparency in Figure 3c & d is adjusted according to the error Δt_0 in the extracted zero-crossing, indicating the confidence level in the measurement. More precisely, the transparency T is determined by the Gaussian distribution $T(\Delta t_0) = 100\% \times \left(1 - \mathrm{e}^{-\frac{\Delta t_0^2}{2\sigma_\Delta^2}}\right)$, with a standard width of $\sigma_\Delta = 8.5$ fs and the transparency values binned into equidistant regions of 5.5 fs starting from 3 fs with respect to the error Δt_0. To exclude areas that exhibit a vastly different instantaneous response, such as those featuring only the bare substrate, we set $T = 100\%$ for each pixel where the maximum in the referenced response $|E_2(t_{\mathrm{EOS}}) - E_2^{\mathrm{MLG}}(t_{\mathrm{EOS}})|$ is larger than 18% of the maximum of the graphene reference $\hat{E}_2^{\mathrm{MLG}}$.

5. Raman characterization of graphene samples

We have performed micro-Raman spectroscopy experiments to estimate the Fermi energies of various graphene samples. Raman spectra are acquired using a Horiba spectrometer, equipped with a $2400\ \mathrm{gr/mm}$ grating, an ×100 objective and a spot size of ~ 1 μm. All spectra are recorded in ambient conditions at an incident wavelength of 532 nm with an incident laser power < 1 mW to avoid any laser heating.

<u>CVD-grown graphene</u>

A typical Raman spectrum from the CVD graphene sample discussed in Figure 2d & e and Figure 3 of the main text is shown in Figure S3a. The G peak position is located at $\mathrm{POS(G)} = 1587.6 \pm 0.3\ \mathrm{cm}^{-1}$ and the 2D peak at $\mathrm{POS(2D)} = 2679.4 \pm 0.1\ \mathrm{cm}^{-1}$. Both peaks are single Lorentzians with $\mathrm{FWHM(G)} = 9.01 \pm 0.1\ \mathrm{cm}^{-1}$ and $\mathrm{FWHM(2D)} = 26.5 \pm 0.3\ \mathrm{cm}^{-1}$, respectively. The 2D to G peak intensity and area ratios are $I(\mathrm{2D})/I(\mathrm{G}) = 1.55 \pm 0.1$ and $A(\mathrm{2D})/A(\mathrm{G}) = 4.48 \pm 0.1$. A negligible small D peak at $\mathrm{POS(D)} = 1350.2 \pm 4.4\ \mathrm{cm}^{-1}$ is visible, with $I(\mathrm{D})/I(\mathrm{G}) < 0.002$. From this data, we extract a Fermi energy of $E_\mathrm{F} \approx 230$ meV, with p-type doping, following the methods outlined in ref 46. Finally, the defect density $n_\mathrm{D} = (5.1 \pm 1.3) \times 10^9\ \mathrm{cm}^{-2}$ is estimated from $I(\mathrm{D})/I(\mathrm{G})$, E_F, and an excitation energy of 2.33 eV (ref 47). We then adopted this technique to investigate the properties of graphene in the scanned sample area presented in Figure 3 of the main text. Figure S3b shows the analysis of the 2D-peak versus G-peak frequency in 15 different positions. The

data points are dispersed along the doping vector,[48] with a spread in POS(G) of approximately 4 cm^{-1}, indicating a quite broad variability of carrier concentration between $\sim 2 \times 10^{12}$ cm^{-2} and 4×10^{12} cm^{-2}, corresponding to E_F between 170 meV and 240 meV (doping calculation is performed using the model in ref 49). We have also studied the presence of defects by analyzing the ratio $I(D)/I(G)$ over the scanned area (Figure S3c & d). In that specific area the D peak is quite intense, with $I(D)$ being a significant fraction of $I(G)$. The resulting density of defects is $n_D \approx 10^{11}$ cm^{-2} and inhomogeneous over the region of interest.

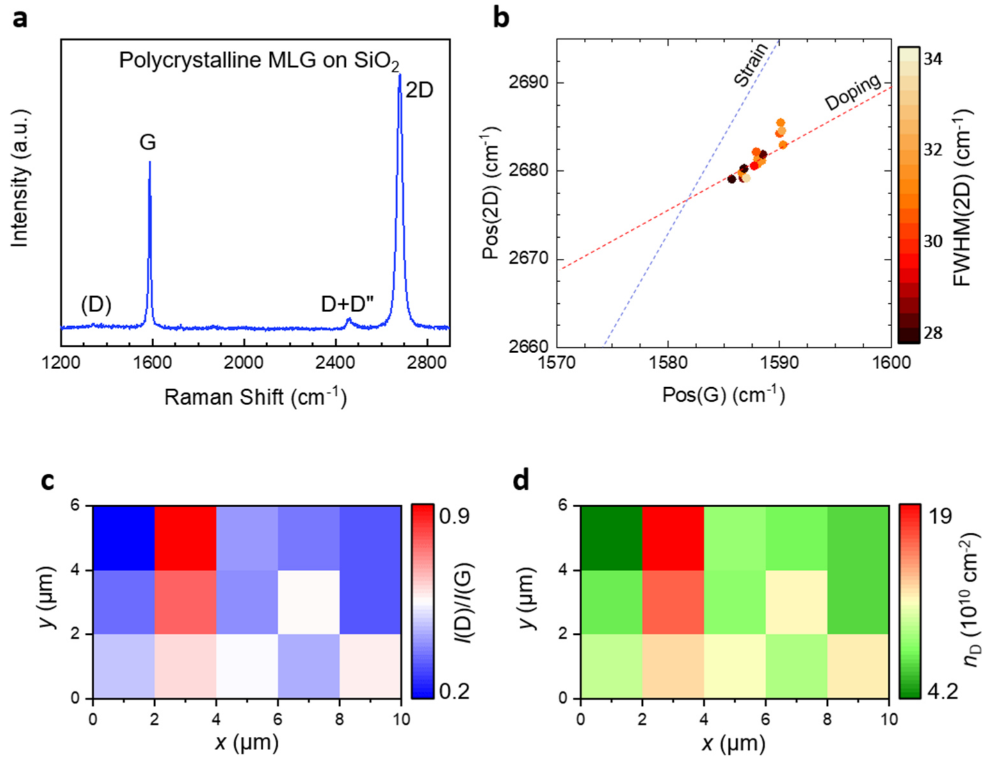

Figure S3. Raman characterization of the CVD graphene sample. a) Typical Raman spectrum at 532 nm of polycrystalline MLG transferred on SiO_2/Si. b) 2D-peak versus G-peak frequency extracted from Raman spectra acquired in different positions on the sample. The color bar represents the values of FWHM(2D) in the recorded spectrum. The dispersion of the data points along the doping axis indicates negligible strain. c, d) Map of the ratio $I(D)/I(G)$ and of the defect density (n_D), measured over the same sample region investigated in the main text (Figure 3).

Dry-exfoliated graphene

Figure S4a shows a typical micro-Raman spectrum from a dry-exfoliated graphene sample, with negligibly small D peak. From the analysis of the frequencies of the G and 2D peaks, we have obtained the POS(2D) vs. POS(G) map displayed in Figure S4b, which allows for a strain-doping vector decomposition.[48,50] Differently from the CVD sample described in the previous section, the exfoliated MLG shows almost uniform doping, with $n \approx 2 \times 10^{12}$ cm^{-2} (ref 50), characterized by a data points dispersion parallel to the strain vector. The sample shows variable tensile strain $\varepsilon_T \approx 0.3\%$.

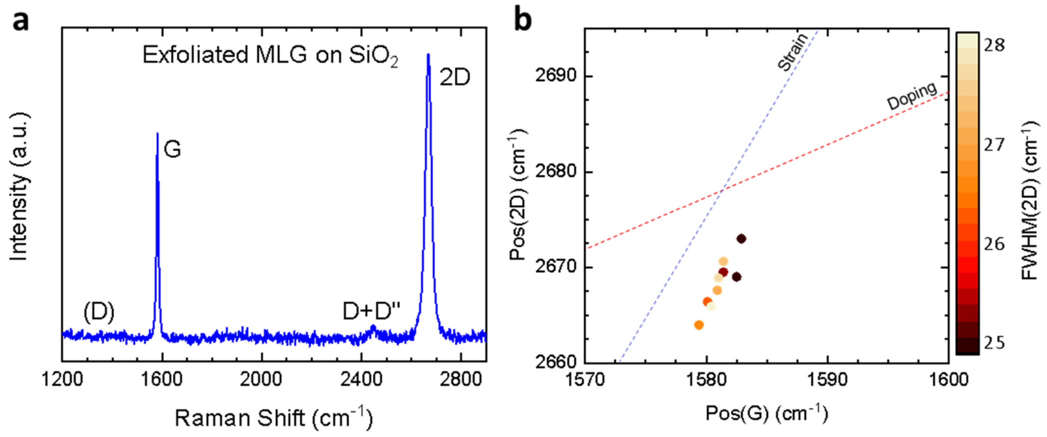

Figure S4. Raman characterization of the exfoliated MLG sample. a) Exemplary Raman spectrum at 532 nm of MLG exfoliated on SiO_2/Si. b) 2D-peak versus G-peak frequency extracted from Raman spectra acquired in different positions on the sample.

6. Impact of Fermi energy, scattering time, and doping of the substrate on the dispersion relation

The shape of the graphene surface polariton dispersion relation depends on several factors, but mostly the Fermi energy, the scattering rate of carriers in graphene and the dielectric surrounding. Varying the Fermi energy moves the polariton branch upwards or downwards, where a change from $E_F = 250$ meV to 100 meV increases the confinement from approximately 10 to 21 at the SPP center frequency of 1.31 THz (left column, Figure S5). Similarly, reducing the scattering time τ from 250 fs to 50 fs increases the confinement factor from approximately 12 to 22, but also leads to a smearing of the dispersion with less prominent features (central column, Figure S5). Finally, increasing the doping

density of the silicon substrate results in an interesting effect called "acoustic surface polariton". The surface mode in graphene can couple to the surface mode on the doped silicon/SiO_2 interface and form a hybrid mode, which has special features like an almost linear dispersion (leading to the defining name "acoustic"). Increasing n_{Si} from 3×10^{15} cm^{-3} to 2×10^{19} cm^{-3} increases the confinement factor from approximately 13 to 26 (right column, Figure S5). An experimental realization of such a system has been demonstrated in ref 19.

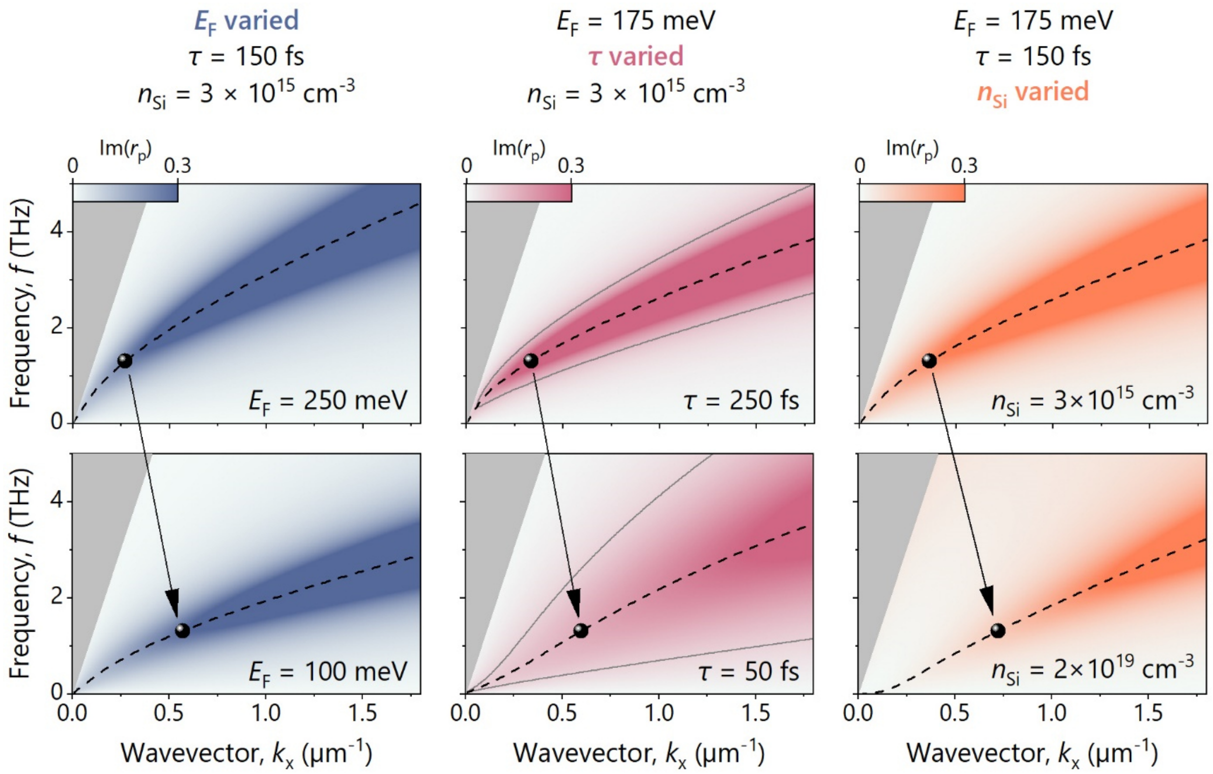

Figure S5. Impact of varying the model parameters on the dispersion relation. Imaginary part of the Fresnel coefficient $r_p(f, k)$, calculated using the transfer matrix method. Reducing the Fermi energy (left column), decreasing the scattering time τ (central column), or increasing the doping n_{Si} (right column) all result in an increased confinement factor k_x/k_{light}. Additionally, reducing the scattering time τ significantly broadens the dispersion relation, as shown by the gray contour lines representing 7% of the maximum value in each map. For a strongly doped substrate, an acoustic plasmon polariton mode emerges, characterized by almost identical phase and group velocities. The black spheres indicate the SPP wave vector at the central frequency of 1.31 THz obtained from fitting the analytical model. The black arrows serve as a guide to the eye to highlight the increase in confinement factor.

7. Evaluating the effect of vacuum annealing on SPP propagation in CVD graphene

We investigated the impact of vacuum annealing on CVD flakes by comparing hypertemporal scans taken before and after the annealing process (150°C for 5 hours at a pressure of $< 10^{-5}$ mbar). Figure S6 shows that no significant changes in polariton propagation are observed as a result of annealing the sample. This is an indication that the scattering rate in our CVD samples is mostly determined by defects rather than surface contaminants like residues originating from the transfer.

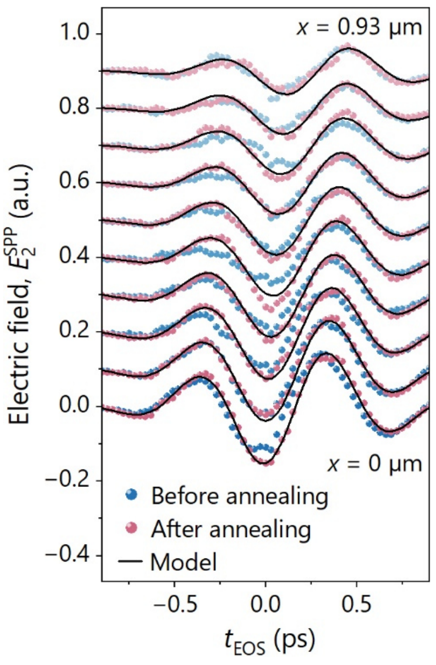

Figure S6. Comparison of polariton propagation before and after annealing a CVD graphene. Experimentally extracted electric field of the SPPs E_2^{SPP} as a function of EOS delay time t_{EOS} for different distances x to the MLG/substrate interface before annealing (blue spheres) and after annealing (red spheres). The black lines correspond to the model evolution with a phase velocity of $v_{\mathrm{ph}} \approx 15.3\ \mu\mathrm{m/ps}$ and a group velocity of $v_{\mathrm{gr}} \approx 13.1\ \mu\mathrm{m/ps}$ (see also Figure S7), which excellently reproduces both data sets.

8. Measurement on a CVD flake with a complementary Fermi energy

Figure S7a shows a hypertemporal map of the surface polariton electric field (after subtracting a reference transient in the same procedure as throughout the manuscript) on a CVD graphene sample with an increased Fermi energy ($E_{\mathrm{F}} \approx 430$ meV, see Figure S8) compared to the sample in Figure 2d ($E_{\mathrm{F}} \approx 230$ meV). Extracting the group and phase velocities from the hypertemporal scans reveals an increased phase velocity as compared to the graphene sample with lower Fermi energy. Plotting phase and group velocity ($v_{\mathrm{ph}} \approx 15.3\ \mu\mathrm{m/ps}$, $v_{\mathrm{gr}} \approx 13.1\ \mu\mathrm{m/ps}$) into a dispersion map and adding the numerically calculated dispersion for the two different Fermi energies (Figure S7b & c) shows that our experimental method perfectly captures the expected theoretical change.

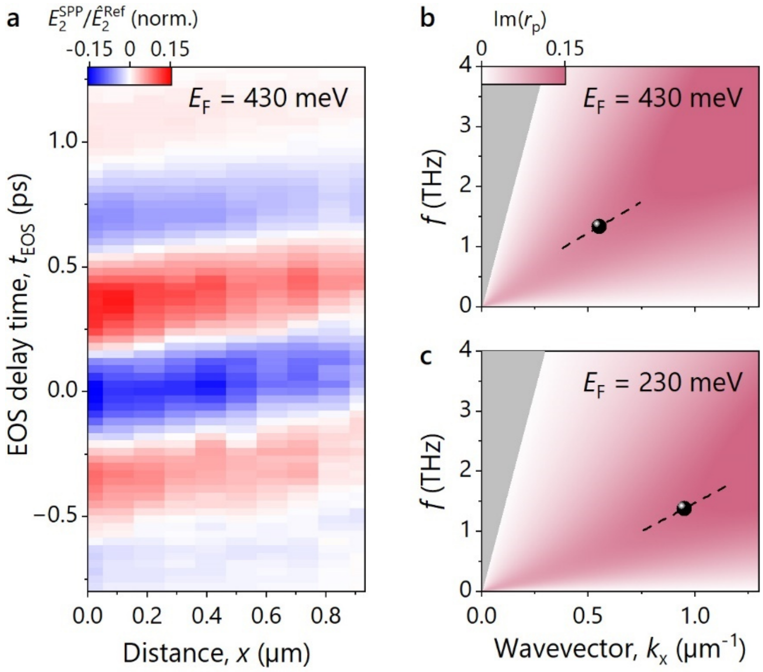

Figure S7. Dispersion relation for different Fermi energies. a) Difference of the hypertemporal map and a reference waveform (not shown), revealing a SPP propagation on a CVD graphene sample with $E_F = 430$ meV. b) Extracted dispersion relation from Figure S7a (black sphere from phase velocity, black dashed line from group velocity) with simulated dispersion relation for $E_F = 430$ meV and $\tau_{CVD} = 23$ fs (magenta background). c) Extracted dispersion relation replotted from Figure 2e of the main text with simulated dispersion relation for $E_F = 230$ meV and $\tau_{CVD} = 23$ fs.

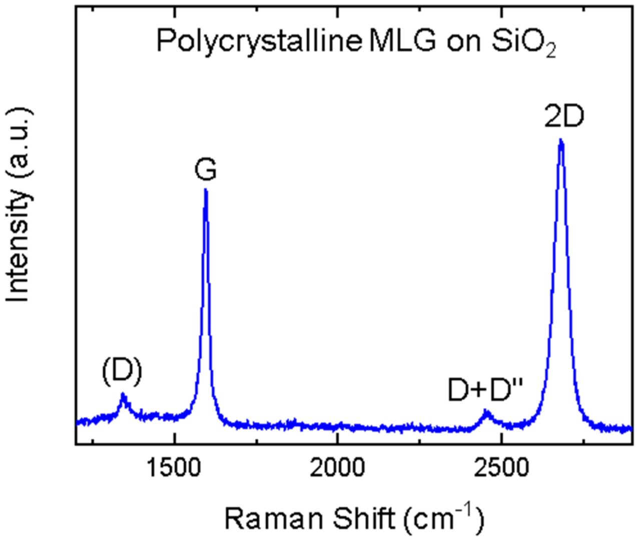

Figure S8. Raman Spectrum of CVD graphene for $E_F = 430$ meV. From the analysis of Raman peaks the following parameters are obtained:

$\mathrm{POS(D)} = 1346.4 \pm 0.7\ \mathrm{cm^{-1}}$,

$\mathrm{POS(G)} = 1595.4 \pm 0.1\ \mathrm{cm^{-1}}$,

$\mathrm{POS(2D)} = 2681.6 \pm 0.1\ \mathrm{cm^{-1}}$,

$\mathrm{FWHM(G)} = 21.2 \pm 0.3\ \mathrm{cm^{-1}}$,

$\mathrm{FWHM(2D)} = 47.4 \pm 0.5\ \mathrm{cm^{-1}}$,

$\mathrm{A(G)/A(2D)} = 0.34$, from which we estimate a Fermi energy of ~430 meV. (details on Fermi energy extraction from Raman spectroscopy, see SI, Section 5)

9. Modeling nonequilibrium surface polariton propagation

In our experiments, the change in propagation of the SPPs upon photoexcitation can be described by replacing the amplitude A_{SPP} in eq 1 of the main text with a time-dependent amplitude $A(t)$ as described by the following formula

$$A(t) = A_{\mathrm{initial}} \times \left[\mathrm{e}^{-\frac{(t-t_{\mathrm{shift}})^2}{2t_{\mathrm{rise}}^2}} \times H(-(t-t_{\mathrm{shift}})) + \mathrm{e}^{-\frac{t-t_{\mathrm{shift}}}{t_{\mathrm{decay}}}} \times H(t-t_{\mathrm{shift}}) \right],$$

where the amplitude $A_{\mathrm{initial}} = 0.22$ reproduces the experimentally observed amplitude change, $H(t)$ is the Heavyside step function, t_{decay} (t_{rise}) is the decay (rise) time, and a shift $t_{\mathrm{shift}} = 295$ fs $- t_{\mathrm{p}}$, which comprises the actual delay between pump and probe, t_{p}, as well as an additional purely mathematical offset of 295 fs. This stems from the difference of the time zero of the experiment and the mathematical description, which is centered at the peak of the pump probe trace. By setting $t_{\mathrm{rise}} = 400$ fs and $t_{\mathrm{decay}} = 2.5$ ps, both in agreement with the evolution of the peak of the pump-induced response as a function of pump delay time (see Figure 4b), we can describe the experimental data from the pumped measurements in Figure 4c, resulting in the modeled $\Delta E_1^{\mathrm{SPP}}$ maps in Figure 4e.

REFERENCES

46. Das, A.; et al. Monitoring dopants by Raman scattering in an electrochemically top-gated graphene transistor. *Nat. Nanotechnol.* **2008**, *3*, 210.

47. Bruna, M.; et al. Doping Dependence of the Raman Spectrum of Defected Graphene. *ACS Nano.* **2014**, *8*, 7432.

48. Lee, J. E.; et al. Optical separation of mechanical strain from charge doping in graphene. *Nat. Commun.* **2012**, *3*, 1024.

49. Stampfer, C.; et al. Raman imaging of doping domains in graphene on SiO_2. *Appl. Phys. Lett.* **2007**, *91*, 241907.

50. Vincent, T.; et al. Probing the nanoscale origin of strain and doping in graphene-hBN heterostructures. *2D Mater.* **2019**, *6*, 015022.
